# Artificial intelligence for endoscopic grading of gastric intestinal metaplasia: advancing risk stratification for gastric cancer

**DOI:** 10.1055/a-2657-9906

**Published:** 2025-09-08

**Authors:** Eduarda Almeida, Miguel Lopes Martins, David Marques, Rose Delas, Tatiana Almeida, Jéssica Chaves, Diogo Libânio, Francesco Renna, Miguel Tavares Coimbra, Mário Dinis-Ribeiro

**Affiliations:** 1 Instituto de Engenharia de Sistemas e Computadores, Tecnologia e Ciência – INESC TEC, Porto, Portugal; 2 Faculdade de Ciências da Universidade do Porto (FCUP), Porto, Portugal; 3 52876 Département STIC, École Nationale Supérieure de Techniques Avancées Bretagne, Brest, France; 4 Precancerous Lesions and Early Cancer Management Group, Research Center of IPO Porto (CI‐IPOP)/CI‐IPOP@RISE (Health Research Group), Portuguese Institute of Oncology of Porto (IPO Porto)/Porto Comprehensive Cancer Center (Porto.CCC), Porto, Portugal; 5 Gastroenterology Department, Portuguese Institute of Oncology of Porto, Porto, Portugal

## Abstract

**Background:**

The Endoscopic Grading of Gastric Intestinal Metaplasia (EGGIM) classification correlates with histological assessment of gastric intestinal metaplasia and enables stratification of gastric cancer risk. We developed and evaluated an artificial intelligence (AI) approach for EGGIM estimation.

**Methods:**

Two datasets (A and B) with 1280 narrow-band imaging images were used for per-image analysis. Still images with manually selected patches of 224 × 224 pixels, annotated by experts, were used. Dataset A was retrospectively collected from clinical routine; Dataset B (used for per-patient analysis) was prospectively collected and included 65 fully documented patients. To mimic clinical practice, a deep neural network classified image patches into three EGGIM classes (0, 1, 2) and calculated the total per-patient EGGIM score (0–10).

**Results:**

On per-image analysis, an accuracy of 87% (95%CI 71%–100%) was obtained. Per-patient EGGIM estimation had an average error of 1.15 (out of 10) and showed 88% (95%CI 80%–96%) accurate clinical decisions for surveillance (EGGIM ≥5), with 85% (95%CI 75%–94%) specificity, no false negatives, and positive and negative predictive values of 62% (95%CI 32%–92%) and 100% (95%CI 100%–100%), respectively.

**Conclusions:**

EGGIM was estimated with high accuracy using AI tools in endoscopic image analyses. Automated assessment of EGGIM may provide a greener strategy for gastric cancer risk stratification, prospective studies, and interventional trials.

## Abstract (de)

**Graphical Abstract:**

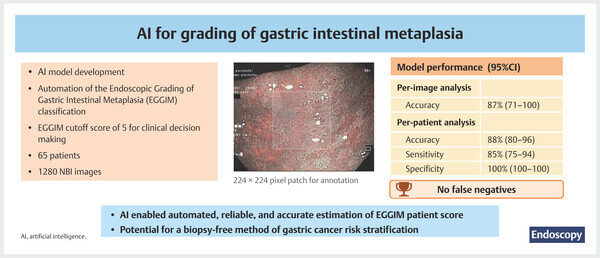

## Introduction

Gastric cancer is the fifth most diagnosed cancer and the fifth leading cause of cancer death globally 1 . Gastric intestinal metaplasia (GIM) is a precancerous condition 2 and individuals harboring GIM are at increased risk of gastric cancer. Its diagnosis involves a widely applied strategy of secondary prevention that relies on close surveillance of high-risk patients, aiming at early gastric cancer diagnosis for improved survival rates 3 .

In fact, GIM is commonly diagnosed through esophagogastroduodenoscopy, which also allows the collection of biopsy samples to effectively confirm the presence of GIM and to allow histological stratification 3 . However, gastric biopsies are among the practices that contribute most to waste production in a hospital 4 5 .

The Endoscopic Grading of Gastric Intestinal Metaplasia (EGGIM) classification allows individual endoscopic risk stratification of gastric cancer by estimating the extent of GIM in a patient’s stomach using image-enhanced technology of virtual chromoendoscopy such as narrow-band imaging (NBI) 6 . This classification system has demonstrated consistency with histological assessment of GIM and value in gastric cancer risk stratification 3 6 7 8 9 , as patients with an EGGIM score ≥5 can be accurately referred for surveillance 6 . As a result, EGGIM has been recommended as a safe and less expensive method of gastric cancer surveillance, with minimal environmental impacts, although specialized training in its use is required 9 . Therefore, the EGGIM classification is likely to benefit from the fast-growing field of artificial intelligence (AI), potentially resulting in a reliable and unbiased tool for estimating per-patient scores. Implementing an AI system for EGGIM scoring, following strict and transparent technical and clinical guidelines 10 , has the potential to provide more accurate and reproducible scores, increasing the likelihood of mass adoption as standard practice. Such a scenario would significantly decrease the need for biopsies.

In this work, we aimed to develop and evaluate an AI approach to EGGIM estimation using NBI images.

## Methods

### Data acquisition

Overall, two curated datasets of NBI images were used for this work: Dataset A (n = 414 images) was collected from a retrospective dataset of medium-resolution images from clinical routine; and Dataset B (n = 866 images) was collected from a prospective dataset of high-quality images from 80 consecutive patients in a controlled environment.

For Dataset A, all images collected from patients scheduled for upper gastrointestinal endoscopy at the IPO-Porto, between December 2019 and September 2020, during regular clinical practice were considered for inclusion in the study, irrespective of clinical indication. Frames considered out of focus, blurred, or showing food or foam were excluded from the analysis. Frames included in this study were those showing the complete spectrum of changes from normal mucosa, atrophic changes, and GIM in the anatomical areas evaluated for EGGIM calculation – lesser curvature of the antrum, greater curvature of the antrum, incisura, lesser curvature of the corpus, and greater curvature of the corpus. For Dataset B, 80 consecutive patients observed in 2024 were considered. Each of them was required to have at least a complete set of five images enabling EGGIM to be estimated. When possible, for both lesser and greater curvatures of the corpus, two different frames (anteversion and retroflexion), for each location, were analyzed for scoring. In total, 65 patients presented full documentation of the five locations.

Endoscopies were performed by experienced gastroenterologists who had each performed over 500 endoscopies per year in the previous 5 years using Olympus high-definition endoscopes (185º or 190º series; Olympus, Tokyo, Japan) equipped with NBI. Images were stored in JPG format with 24-bit color depth and resolution of 640 × 480 pixels.

Both datasets contributed to the per-image analysis, and Dataset B provided preliminary assessment for a per-patient EGGIM estimation.

### Labeling, annotation, and outcomes

To achieve optimal image framing, a 224 × 224 pixel visual patch was manually selected for each image according to all three of the following criteria, mimicking current recommendations for acquiring endoscopic images when performing biopsies at these sites:

1. prioritize the center of the image
2. avoid medical devices (such as endoscope), and
3. ensure representativeness of the anatomical location.

**Fig. 1:**
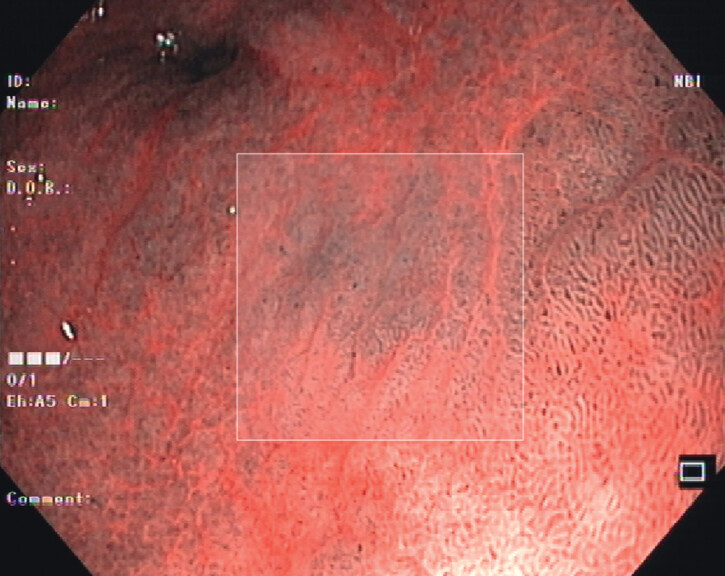
Example of a 224 × 224 pixel image patch selected from an esophagogastroduodenoscopy image acquired using narrow-band imaging. The anatomical location represented in the image is the greater curvature/posterior wall of the proximal antrum and the Endoscopic Grading of Gastric Intestinal Metaplasia classification score represented is 2.

An example of the manual patch selection is presented in Fig. 1 .

Initial image annotation was carried out by a trained nonclinical expert who benefited from close guidance of two experienced gastroenterologists, and using the SuperAnnotate annotation platform (SuperAnnotate AI, Sunnyvale, California, USA). Moreover, manual annotation was performed retrospectively by reviewing still images collected during esophagogastroduodenoscopy, and annotators were blinded to histopathological findings. Image labels included anatomical location and EGGIM classification for each specific patch, as well as a final clinical decision for each patient in Dataset B according to total EGGIM score. For this manual annotation, expert and nonexpert annotators followed the taxonomical descriptions previously described as guidance 11 12 13 .

The EGGIM classification of each patch attributed by the AI system was compared with the classification determined by clinical experts to evaluate the performance of the model, both on per-image and per-patient analyses. The extent of GIM in each of the five different anatomical locations of the stomach was determined by differentiating between EGGIM classes of 0, 1, or 2 (defined as no metaplasia, focal [≤30%] metaplasia, and extensive [>30%] metaplasia, respectively), while the overall extent of GIM in each patient was obtained by summing up the EGGIM scores at each anatomical location, thus estimating the final EGGIM per patient (0–10).

### Experimental set-up

The algorithm developed for the automated estimation of the score used a leave-one-patient-out cross-validation approach to test the model, making sure that data from each individual patient remained entirely in a single fold, totaling 65 folds. The remaining data was split per each fold, with 90% being used for training and 10% for both validation and early-stopping of the model.

### Algorithm architecture

A deep neural network (DNN) was trained, using combined data from both datasets, for classification of patches into three EGGIM classes (0, 1, 2). Specifically, we used a ResNet-50 as a fixed backbone, given its superior performance reported by He et al. 14 and previous successful application to endoscopic images 15 , coupled with global average pooling. Overall, the EGGIM classification in a patch was surmised as a task analogous to a fine-grained texture classification 11 . The leave-one-patient-out (65 folds) cross-validation strategy allowed better estimates of the variance of these architectures and fine-tuning strategies given the limited training data. Therefore, the training set was augmented via stratified sampling of Dataset A applying the empirical class statistics of data selected for training from Dataset B. The fine-tuning strategies consisted of, for each fold, training the model using the Adam gradient descent algorithm to minimize the weighted cross-entropy loss, and applying early stopping in the validation set to mitigate class imbalance and sampling bias. Our implementation code is available on request. Results of this step were then combined with images from Dataset B to obtain a 0–10 per-patient EGGIM estimation.

### Analyses

A descriptive statistical analysis was performed using absolute and relative frequencies. The accuracy was assessed for both per-image and per-patient analyses, while the specificity and sensitivity were measured for per-class and per-patient analyses. Moreover, the mean absolute error, as well as the positive and negative predictive values were also determined for the per-patient analysis. These outcome measures, reported with 95%CI, were based on and compared with the current guidelines 3 , as well as with previous reports of EGGIM scoring performances 6 7 8 9 . These publications established an EGGIM cutoff score of 5 on which to base individual risk stratification and subsequent clinical decision. All analyses were carried out using Python v3.9.6, integrated with SciPy v1.15.2, Seaborn v0.12.2, and Matplotlib v3.10.1 (Python Software Foundation, Wilmington, Delaware, USA) and Microsoft Excel v Office 365 (Microsoft, Redmond, Washington, USA).

The DNN-based EGGIM risk model was tested for three different prevalence scenarios (5%, 25%, 50%) to determine probabilities of diagnosing or not diagnosing extensive GIM. These values were calculated for positive and negative scenarios and applying Bayes’ theorem to the DNN’s sensitivity and specificity. Biopsy recommendations were determined accordingly.

The structure, pipeline (see **Fig. 1s** in the online-only Supplementary material), and research options selected for this work followed Quality Assessment of pre-clinical AI studies in Diagnostic Endoscopy (QUAIDE) guidelines 10 .

## Results

### Per-image analyses

**Fig. 2:**
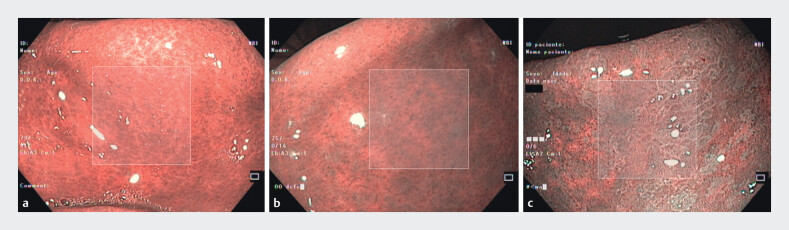
Different extents of intestinal metaplasia in the lesser curvature of the antrum using the Endoscopic Grading of Gastric Intestinal Metaplasia (EGGIM) classification. **a** EGGIM0 (no metaplasia). **b** EGGIM1 (focal [≤30%] metaplasia). **c** EGGIM2 (extensive [>30%] metaplasia). The 224 × 224 pixel image patch selection is also indicated.

**Fig. 3:**
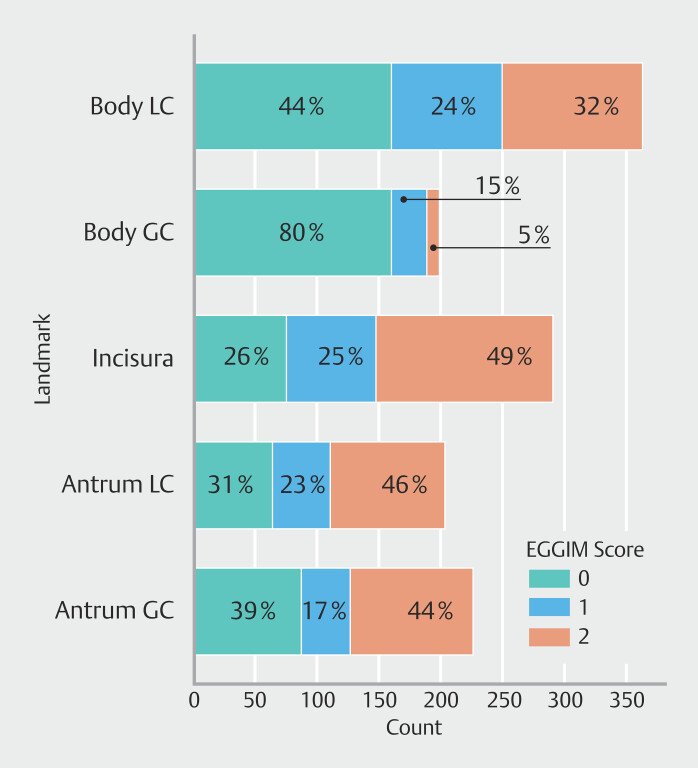
Endoscopic Grading of Gastric Intestinal Metaplasia (EGGIM) class distributions across anatomical locations in the stomach by EGGIM score (0, 1, or 2) per stomach anatomical location. EGGIM0, no metaplasia; EGGIM1, focal (≤30%) metaplasia; EGGIM2, extensive (>30%) metaplasia; GC, greater curvature; LC, lesser curvature.

From Dataset B, 866 NBI images from 80 patients were used for the DNN validation, which showed an accuracy of 87% (95%CI 71%–100%) for the per-image prediction of the EGGIM score. Moreover, the DNN showed GIM detection capability, by differentiating between classes EGGIM0 vs. EGGIM1+EGIMM2 with high sensitivity, specificity, and overall accuracy of 79%, 94%, and 85%, respectively ( Table 1 ). The extent of GIM (EGGIM0+EGGIM1 vs. EGIMM2) was also reliably predicted by the DNN, with 96% sensitivity, 84% specificity, and 86% accuracy ( Table 1 ). Fig. 2 shows representative examples of images and patches with different EGGIM levels that were used in the study. Fig. 3 shows the overall distribution of EGGIM scores per location.

**Table:** **Table 1** Deep neural network model performance metrics on per-image analyses, using leave-one-patient-out cross-validation.

	EGGIM0 vs. EGGIM1+EGIMM2 ^1^	EGGIM0+EGIMM1 vs. EGGIM2 ^1^
Performance, % (95%CI)		
Sensitivity	79 (75–83)	96 (92–99)
Specificity	94 (92–97)	84 (81–87)
Accuracy	85 (83–88)	86 (84–89)
EGGIM, Endoscopic Grading of Gastric Intestinal Metaplasia. ^1^ EGGIM0, no metaplasia; EGGIM1, focal (≤30%) metaplasia; focal metaplasia; EGGIM2, extensive (>30%) metaplasia.		

### Per-patient analyses

The final per-patient EGGIM estimation in Dataset B, using DNN predictions, achieved a mean absolute error of 1.15 (out of 10) when compared with clinical experts’ evaluations, which resulted from 8 incorrect predictions out of 65 patients ( Table 2 , **Table 1s** ). The model provided 88% (95%CI 80%–96%) correct clinical decisions on individual risk stratification (accuracy), which were based on the previously established threshold of EGGIM ≥5 for surveillance ( Table 3 ). The incorrect decisions consisted of 8 false positives and 0 false negatives ( Table 2 , Table 3 , **Table 1s** ). As such, none of the patients requiring follow-up care would be ruled out.

**Table:** **Table 2** Overall performance of the deep neural network in the clinical decision-making process. 2 × 2 performance table.

	Clinical assessment		DNN prediction total
	EGGIM <5, n	EGGIM ≥5, n	
DNN predictions			
EGGIM <5, n	44	0	44
EGGIM ≥5, n	8	13	21
Clinical assessment total	52	13	65
DNN, deep neural network; EGGIM, Endoscopic Grading of Gastric Intestinal Metaplasia.			

**Table:** **Table 3** Overall performance of the deep neural network in the clinical decision-making process. Performance metrics.

DNN performance (95%CI)	
Sensitivity, %	100 (100–100)
Specificity, %	85 (75–94)
Accuracy, %	88 (80–96)
Positive likelihood ratio	6.5000 (3.4362–12.2954)
Negative likelihood ratio	0.0000 (0.0000–0.0000)
Positive predictive value, %	62 (32–92)
Negative predictive value, %	100 (100–100)
DNN, deep neural network; EGGIM, Endoscopic Grading of Gastric Intestinal Metaplasia.	

Moreover, the estimated probabilities for the presence of extensive GIM and associated biopsy recommendations were obtained for different prevalence scenarios, and showed that no biopsies were needed when the model estimates extensive EGGIM as negative ( Table 4 ).

**Table:** **Table 4** Comparison of predictive values for gastric intestinal metaplasia and corresponding need for biopsies estimated according to different prevalences of gastric intestinal metaplasia obtained with the deep neural network model, when considering the Endoscopic Grading of Gastric Intestinal Metaplasia classification cutoff score of ≥5.

Prevalence	Estimated chance of extensive GIM if DNN estimates EGGIM ≥5, %	Biopsies	Estimated chance of extensive GIM if DNN estimates EGGIM <5, %	Biopsies
5% (low-prevalence countries)	24	Yes	0	No
25% (high-prevalence settings)	62	Yes	0	No
50% (patients under follow-up)	87	No	0	No
DNN, deep neural network; EGGIM, Endoscopic Grading of Gastric Intestinal Metaplasia.				

## Discussion

As far as we are aware, this is the first study applying AI to determine the final clinical approach for individuals with precancerous conditions in the stomach, as well as the first to do so following the latest standards established for reporting such studies, namely QUAIDE 10 .

A DNN-based system was deployed and exhibited a very interesting overall performance in automated EGGIM scoring of image patches, thus highlighting the ability of this AI system to spot key features of GIM, such as complex and fine-grained textures. The use of still images and patches effectively mimics the clinical approach during an esophagogastroduodenoscopy, as still images are commonly frozen and used to estimate the EGGIM score, and visual central focus is largely used to guide biopsy targeting when necessary.

In per-image analyses, the system achieved good sensitivity, both for GIM detection (EGGIM0 vs. EGGIM1+EGGIM2) and for assessment of GIM extent (EGGIM0+EGGIM1 vs. EGGIM2), in congruence with an AI-based system previously evaluated for direct diagnosis of GIM (i.e. not assessing EGGIM score), which showed 95% sensitivity 16 . When considering the EGGIM classification criteria that suggest individual scores ≥5 can be accurately referred for clinical surveillance without the need for biopsies 6 , the DNN model was able to provide per-patient analyses, achieving 88% accuracy and 100% sensitivity in clinical decision making based on this threshold, with a good specificity. These results were achieved even with datasets enriched with extensive GIM cases ( **Fig. 3** ), which are predominant in oncology hospitals such as IPO-Porto, but represent a clinically significant and complex scenario.

In this initial preclinical study, none of the patients who required surveillance would be excluded as there were no false-negative results. This 100% sensitivity shows that the DNN model surpassed prior AI tools used for the direct diagnosis of GIM (95% sensitivity), as well as endoscopists assessments (79% sensitivity) 16 , even though the specificity of this model (85%) was lower than that of endoscopists (90%) 16 . The low mean absolute error in EGGIM score prediction in the per-patient analysis, compared with manual annotations, suggests that this AI system approximates human performance closely. Notably, these results were achieved even when a significant percentage of the training data were from Dataset A, which had very different characteristics from the test dataset (Dataset B), suggesting strong generalization potential. Additionally, the model showed that an increasing chance of diagnosing extensive GIM correlated with increasing prevalence of positive results (24% at low, 62% at mid, and 87% at high prevalence), with 0% for negative results ( Table 4 ). Esposito et al. 6 reported estimated values of 15.3% at low prevalence, 46.5%–55.6% at mid prevalence, and 84.6%–88.8% at high prevalence. Therefore, the DNN is more conservative when evaluating extensive GIM risk compared with Esposito et al. 6 but allows biopsies to be avoided when significant GIM is not determined ( Table 4 ).

Altogether, these findings emphasize the robustness of DNN technology for EGGIM scoring and highlight its potential for high-stakes clinical applications, ultimately positioning this study as a key breakthrough in the field of endoscopic diagnostics assisted by AI. While the original EGGIM system works well for experts 6 , less experienced endoscopists can benefit considerably from the model proposed, as it can enable them to use the EGGIM score with greater confidence and, consequently, reduce the need for biopsies. Moreover, all practitioners would also benefit from the standardization and automation of the EGGIM system achieved with the AI system, thus ensuring not only consistency, but also an objective EGGIM classification.

Beside the clinical application, the automated EGGIM classification has great potential to be implemented in longitudinal studies, by facilitating the follow-up of individual variations in GIM extent over time. This AI model might also be used in cross-sectional comparisons to allow a more direct and unbiased comparison across clinical studies and different virtual chromoendoscopy technologies. Similarly, this model might be applied across different ethnicities, populations, and clinical environments. Although gastric cancer incidence can vary dramatically between ethnicities or populations 17 , endoscopic visual features of the gastric mucosa are not expected to present differences between these groups. However, distinct clinical experts and environments might embed technical differences in images, such as quality and lighting, which may impact markedly the performance of AI models. Further research targeting these nuances, as well as the enumerated longitudinal and cross-sectional applications of the automated EGGIM scoring model will allow assessment of its generalizability and impact.

Despite these encouraging results, some limitations must also be addressed. Manual annotations of images from the 65 patients in Dataset B were not validated by histological studies, the real generalizability power of this model remains unclear (namely to other centers or providers of virtual chromoendoscopy), and the lower specificity compared with endoscopist evaluation may result in unnecessary follow-ups. Furthermore, real-time implementation during live endoscopy has not been explored.

Overall, the DNN-based EGGIM scoring system showed potential for clinical incorporation, as a reliable and automated decision-making support tool for stratifying gastric cancer risk and personalizing individual follow-up care.

In the near future, the use of virtual chromoendoscopy with AI, as in the automated EGGIM estimation tool described here, will offer endoscopists noninvasive methods of evaluating GIM, both with good accuracy and above the threshold of 90% sensitivity established for clinical relevance 18 . Ultimately, this might lead to a reduced need for biopsies, aligning with the economic and clinical perspectives presented by Libânio et al. 19 , and providing additional advances toward a greener strategy in gastric cancer risk stratification and surveillance.

## Supplementary Material

Supplementary Material Zusatzmaterial
