# Supplementary material for: Artificial intelligence for endoscopic grading of gastric intestinal metaplasia: advancing risk stratification for gastric cancer
Source: Endoscopy. 2025 Sep 8;57(11):1254–60. doi: 10.1055/a-2657-9906 (PMC12599144; doi:10.1055/a-2657-9906)

Supplementary Material

Eduarda Almeida, Miguel L. Martins, David Marques, Rose Delas, Tatiana Almeida, Jéssica Chaves, Diogo Libânio, Francesco Renna, Miguel Coimbra, Mário Dinis-Ribeiro

Artificial intelligence for endoscopic grading of gastric intestinal metaplasia: advancing risk stratification for gastric cancer

Table 1s Comparison between EGGIM scores provided by clinical experts vs. predicted by the DNN model for the 65 patients in the Dataset-B (1 to 65).

Patient	Patient final EGGIM score evaluated by clinical experts [a]	Patient EGGIM score predicted by the model
**1**	1	4
**2**	3	8[#]
**3**	1	6[#]
**4**	8[#]	8[#]
**5**	5[#]	6[#]
**6**	2	2
**7**	0	0
**8**	0	0
**9**	1	1
**10**	8[#]	8[#]
**11**	8[#]	9[#]
**12**	1	3
**13**	7[#]	10[#]
**14**	3	5[#]
**15**	1	1
**16**	2	4
**17**	6[#]	6[#]
**18**	0	5[#]
**19**	0	0
**20**	4	4
**21**	2	3
**22**	7[#]	7[#]
**23**	2	2
**24**	0	4
**25**	2	2
**26**	2	7[#]
**27**	5[#]	8[#]
**28**	1	0
**29**	6[#]	6[#]
**30**	3	3
**31**	1	0
**32**	0	0
**33**	0	1

Patient	Patient final EGGIM score evaluated by clinical experts [a]	Patient EGGIM score predicted by the model
**34**	0	2
**35**	3	5[#]
**36**	0	0
**37**	6[#]	7[#]
**38**	2	3
**39**	2	2
**40**	8[#]	8[#]
**41**	1	2
**42**	4	4
**43**	1	2
**44**	0	6[#]
**45**	0	0
**46**	1	1
**47**	0	2
**48**	1	0
**49**	2	2
**50**	4	4
**51**	0	0
**52**	1	1
**53**	0	0
**54**	3	5[#]
**55**	4	2
**56**	1	1
**57**	0	0
**58**	0	1
**59**	0	0
**60**	1	1
**61**	5[#]	8[#]
**62**	4	4
**63**	0	0
**64**	6[#]	5[#]
**65**	2	1

[a] The EGGIM scores manually annotated were determined based on the selected 224*224 patches.

[#] Patients signalized for surveillance based on EGGIM score threshold of ≥ 5.

Patients highlighted in grey represent false positives predicted by the DNN model.

Fig. 1s Scheme illustrating the overall pipeline followed in this work, from data processing and model training to EGGIM scoring and clinical decision making.

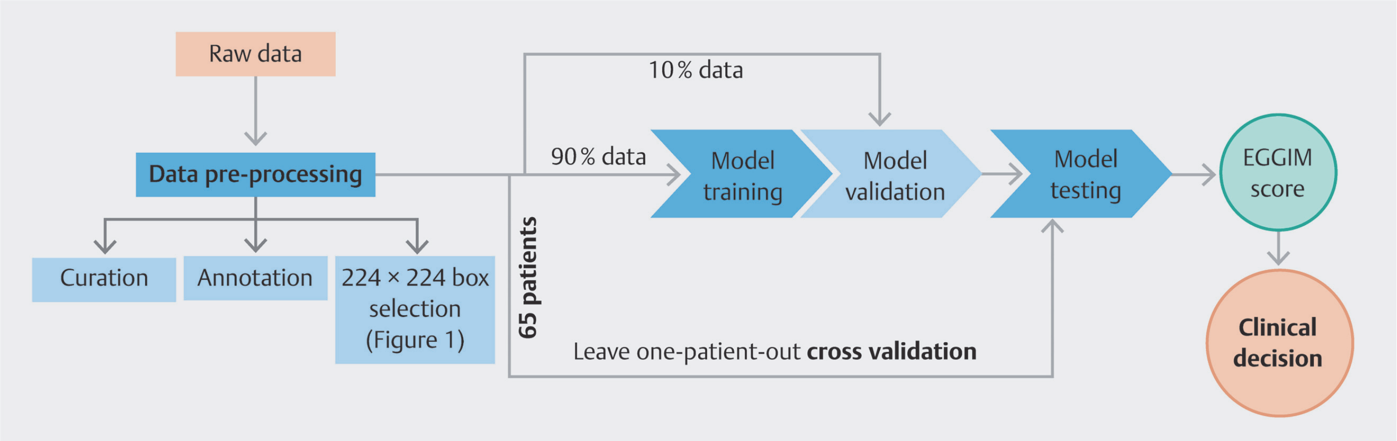

Supplement: Supplementary file 1 — Supplementary Material [file 10-1055-a-2657-9906_26920540.pdf]
